# Lifetime sexual violence among transgender women and travestis (TGW) in Brazil: Prevalence and associated factors

**DOI:** 10.1590/1980-549720240013.supl.1

**Published:** 2024-08-19

**Authors:** Bruna Hentges, Rafael Steffens Martins, Jonatan da Rosa Pereira da Silva, Dariana Pimentel Gomes Hübner, Andréa Fachel Leal, Luciana Barcellos Teixeira, Daniela Riva Knauth, Maria Amélia de Sousa Mascena Veras

**Affiliations:** I Universidade Federal do Rio Grande do Sul, Postgraduate Program in Epidemiology –Porto Alegre (RS), Brazil.; II Universidade Federal do Rio Grande do Sul, Department of Sociology – Porto Alegre (RS), Brazil.; III Universidade Federal do Rio Grande do Sul, Department of Public Health – Porto Alegre (RS), Brazil.; IV Santa Casa de São Paulo, School of Medical Sciences – São Paulo (SP), Brazil.

**Keywords:** Transgender women, Sexual violence, Social vulnerability.

## Abstract

**Objective:**

To describe the prevalence, characteristics, and factors associated with sexual violence in transgender women and *travestis* (TGW) in Brazil.

**Methods:**

This cross-sectional study was conducted in five Brazilian cities (Campo Grande, Manaus, Porto Alegre, Salvador, and São Paulo) between 2019 and 2021. Participants were recruited using the respondent-driven sampling (RDS) technique. The outcome of interest is the self-reported experience of sexual violence throughout the respondents’ lifetime. We evaluated the actions taken by victims of sexual violence and how they dealt with the experience. Logistic regression analysis was employed to examine the associations between sociodemographic and behavioral factors (such as race, income, drug use, sex work, and access to healthcare) and the outcome.

**Results:**

A total of 1,317 TGW were interviewed. Among them, 53% (n=698) reported experiencing sexual violence. For 64.4% (n=419) of the respondents, sexual violence occurred on more than one occasion. The majority of TGW did not seek health services (93.2%, n=648), disclose the violence (93.9%, n=653), nor seek support from family or friends (86.5%, n=601). A higher prevalence of sexual violence was associated with homelessness (adjusted prevalence ratio — aPR=1.69, 95% confidence interval — 95%CI 1.01–2.84), a history of engaging in sex work (aPR=2.04, 95%CI 1.46–2.85), self-reporting regular, bad, or very bad emotional health (aPR=1.67, 95%CI 1.28–2.19), and experiencing difficulties accessing health services in the previous year (aPR=2.78, 95%CI 1.74–4.43).

**Conclusion:**

The high prevalence of sexual violence, analyzed together with the actions of the victims, indicates a context of high vulnerability and low institutional support. In this scenario, violence can be exacerbated, resulting in severe health consequences.

## INTRODUCTION

Sexual violence is a violation of human rights, with profound physical, psychological, and social consequences for its victims. *Sexual violence* is an umbrella term that includes different types of coercion, from social pressure and intimidation to physical force and forced penetration^1^. Among cisgender people (individuals whose gender aligns with their sex at birth), sexual violence affects women proportionally more, with an estimated lifetime prevalence of violence of 35.6%^1^.

For transgender individuals, those whose gender differs from the one assigned at birth^2^, existing studies consistently indicate a higher prevalence of sexual violence, along with other forms of violence. In the 2015 US Transgender Survey (USTS), the most extensive study conducted with transgender individuals to date (n=27,715), 47% of all respondents (transgender men and women) reported experiencing sexual violence during their lifetime^3^. Specifically for transgender women, the lifetime prevalence of sexual violence varies across different regions of the world: in the USTS, the prevalence for this group was 37%^3^; in a study encompassing five African countries, it reached 54.4%^4^; and, in a study conducted in Haiti, the prevalence was reported as 75.5%^5^.

The number of studies investigating this phenomenon has substantially increased in recent years. However, a significant proportion of them examines transgender individuals as a singular group, without distinguishing between transgender men and women or even incorporating them under the umbrella of the “LGBT” identity. This approach tends to homogenize the experiences of transgender men and women, obscuring the various psychosocial stressors unique to each gender identity^6^. In this regard, the present study aims to describe the prevalence, characteristics, and factors associated with sexual violence in a sample of transgender women and *travestis*.

Transgender women frequently fall victim to sexual violence as a consequence of their gender identity and expression, which is perceived as deviating from societal norms and expectations^7,8^, challenging the established power dynamics of a heteronormative society^9^. In regions lacking specific legislation to safeguard this population, instances of sexual violence are exacerbated. Seeking assistance often leads to increased discrimination and violence from health professionals^7,10^ and law enforcement^11,12^, amplifying the challenges faced by transgender women.

Violence, in its different forms, exerts an adverse influence on the health and well-being of transgender women, not only through immediate physical harm but also by intensifying psychological suffering^6^. Studies have shown that violence perpetrated against transgender women is associated with different adverse health outcomes, such as depressive disorders^13^, anxiety^14^, suicidal ideation^15^, and use of illegal substances^16^. Sexual violence adds even more vulnerability^17^ to the lives of transgender women, which is already permeated by a complex chain of social and health inequities.

Violence in its most severe manifestation, homicide, exhibits a concerning prevalence within the context of Latin America. Non-governmental organizations within the LGBTQIA+ community, dedicated to monitoring global statistics on violence perpetrated against transgender individuals, report that 73% of such homicides occur within Latin American territories^18^. Brazil, specifically, emerges as the primary locus of transgender homicide incidents, being responsible for 31% of all such occurrences worldwide during the period spanning 2022 to 2023^18^. In this context, it is necessary to understand the extent of the problem of violence perpetrated against transgender women and *travestis* in the country.

In the Latin American region, the term “transvestite” (*travesti*, in Portuguese) is often adopted by transgender women whose trajectories are characterized by extreme vulnerability, with prostitution being the main form of subsistence^19^. In order to preserve the political contextualization and not neglect the history of social and economic marginalization faced by transvestites, the present study will choose to use the term “transgender women and *travestis*” (TGW) to refer to the group under analysis.

## METHODS

The data analyzed in this article were obtained from the cross-sectional research entitled “Study on the Prevalence of Syphilis and other Sexually Transmitted Infections among Transvestites and Transexual Women in Brazil: Care and Prevention” (TRANS Odara), carried out in five Brazilian capitals: Porto Alegre, São Paulo, Campo Grande, Salvador, and Manaus. As this group is complex to access using traditional sampling techniques, the respondent-driven sampling (RDS) technique was used, according to which the participants invited people from their network of relationships to participate in the study^20^.

The eligibility criteria established for participation in the study were: being 18 years of age or older; assigned male sex at birth; identifying as a transvestite, transgender woman, or any other self-designation that implied a transfeminine gender identity; living, working or studying in the cities included in the scope of the study; and presenting a referral coupon provided by a known recruiter, following the RDS sampling methodology. The exclusion criteria were being under the influence of alcohol or psychoactive substances in such a way that made the interview impossible.

Data collection occurred between December 2019 and July 2021. The study began with a formative phase, utilizing various research methods such as mapping social venues. The seeds of the study were identified through in-depth interviews and focus groups within the transgender women and *travestis* community. A sample size of 1,280 transgender women and *travestis* was determined to estimate syphilis prevalence at each site. Despite pandemic-related challenges hindering on-site training, researchers with prior RDS study experience facilitated online training and supervision for all research activities. Study participants completed a questionnaire covering sociodemographic information, sexual behavior, substance abuse, and experiences of violence. Additionally, participants underwent rapid tests for HIV, syphilis, hepatitis B, and hepatitis C, and provided samples of blood, urine, and secretions for subsequent laboratory tests. Further details about the research design can be found in another previously published article^21^.

### Variables

The outcome of interest in the study is the experience of sexual violence during life. The outcome variable was created based on two distinct questions: whether, at some point in her life, the interviewee was forced to have sex (yes/no) and whether the first sexual intercourse was forced.

Sociodemographic and behavioral variables were used to describe the sample and compare groups. Age was categorized into groups (18 to 29, 30 to 39, and 40 years or more). Race/color was self-declared and considered white, black (including black and brown), and yellow/indigenous. Education was categorized based on years of study: less than eight (corresponding to incomplete primary education), eight to 11 (corresponding to complete primary education/incomplete secondary education), and 12 or more years (complete secondary education or higher). The income variable was categorized based on the Brazilian minimum wage (BMW) in force at the time of the study (R$ 1,045.00, approximately 217.15 dollars). For logistic regression analysis, the following variables were dichotomized: race/color (white and non-white), education (less than 12 years of study and 12 years or more), income (up to one BMW or more than one), and problems in accessing health services in the previous 12 months (yes and no).

Respondents who declared living in their own home, rented home, or temporarily with friends/family were categorized as “having a stable place to live”. Those who declared that they lived on the streets, in a boarding house, or where they worked were considered “not having a place to live.” Commercial sex was assessed based on the question: “Have you ever had sex in exchange for money, goods, drugs, or a place to live?” The variable “sex worker” was constructed from two questions: main occupation at the time of the study and declaration that the primary source of income in the last month came from this occupation. The use of illegal drugs was assessed in the previous twelve months and included marijuana (a drug considered illegal in Brazil). The answers about the health status at the time of the study and the emotional health of the participants were dichotomized. The assessment of difficulties in accessing health services was framed by the following question: “Over the past year, have you encountered any challenges accessing health services related to your gender identity?”.

For interviewees who were victims of sexual violence, we assessed the frequency of sexual violence (once or more than once) and the perpetrator of the last violence suffered. The perpetrator was categorized as an unknown person, someone from the victim’s daily life (such as a neighbor, attendant, customer, or boss), and an intimate of the victim (friend, family member, or partner). The resources used by the victim after the last violence to deal with what happened were verified: whether they sought any health services, filed a complaint with a formal institution (such as police), and sought support from family or friends. Resources were considered for the “sought any type of help” variable.

Considering that the study outcome is the lifetime experience of sexual violence, we hypothesize that older transgender women and *travestis* may have a higher prevalence of such experiences due to their increased exposure over time. Therefore, we decided to investigate this relationship, as well as whether age correlates with varied patterns of seeking assistance among transgender women and *travestis*. Consequently, we stratified Table 1 by age group to further explore these associations.

**Table 1:** Sociodemographic and behavioral characteristics of transgender women and *travestis* in Brazil, stratified by sexual violence.

Variable	Sexual violence			
	Total sample (n=1,317)	Yes (n=698, 53%)	No (n=619, 47%)	p-value
Age group				
18 to 29	603 (46,5)	322 (53,4)	281 (46,6)	0,379
30 to 39	392 (30,2)	220 (56,1)	172 (43,9)	
=40	301 (23,2)	153 (50,8)	148 (49,2)	
Race/skin color				
White	336 (25,7)	176 (52,4)	160 (47,6)	0,943
Black	924 (70,8)	494 (53,5)	430 (46,5)	
Yellow/indigenous	45 (3,5)	24 (53,3)	21 (46,7)	
Education (years)				
<8	328 (25,0)	182 (55,5)	146 (44,5)	0,025
8 to 11	712 (54,2)	354 (49,7)	358 (50,3)	
=12	273 (20,8)	160 (58,6)	113 (41,4)	
Income (BMW)*				
<1	593 (49,5)	341 (57,5)	252 (42,5)	0,084
1 to 2	424 (35,4)	214 (50,5)	210 (49,5)	
>2	180 (15,1)	97 (53,9)	83 (46,1)	
Living conditions				
Having a stable place to live	1,166 (92,0)	608 (52,1)	558 (47,9)	0,030
Not having a place to live	101 (8,0)	64 (63,4)	37 (36,6)	
Commercial sex (life)				
Yes	947 (73,4)	557 (58,0)	404 (42,0)	<0,001
No	344 (26,6)	141 (39,6)	215 (60,4)	
Sex worker (current)				
Yes	535 (41,4)	324 (60,0)	216 (40,0)	0,001
No	756 (58,6)	374 (48,1)	403 (51,9)	
Illegal drugs (12 months)				
Yes	727 (55,2)	418 (57,5)	309 (42,5)	<0,001
No	590 (44,8)	280 (47,5)	310 (52,5)	
Challenges to access health services (12 months)				
No, and sought	758 (59,1)	392 (51,7)	366 (48,3)	<0,001
No, but didn’t seek	384 (30,0)	191 (49,7)	193 (50,3)	
Yes	140 (10,9)	106 (75,7)	34 (24,3)	
Self-declared health status				
Good/very good	877 (68,6)	452 (51,5)	425 (48,5)	0,015
Regular/bad/very bad	401 (31,4)	236 (58,9)	165 (41,1)	
Self-declared emotional health				
Good/very good	650 (51,1)	304 (46,8)	346 (53,2)	<0,001
Regular/bad/very bad	623 (48,9)	379 (60,8)	244 (39,2)	

Differences in numbers are due to missing values. BMW: Brazilian minimum wage.

* Brazilian minimum wage at the time (R$ 1,045.00, approximately 217.15 dollars).

### Statistical analysis

Analyses were conducted utilizing the Statistical Package for Social Sciences (SPSS, version 22.0) software. The sample characteristics were delineated by category N and percentage. Group comparisons employed Pearson’s χ² homogeneity test. Factors associated with sexual violence were analyzed by bivariate and multivariable models that estimated the prevalence ratios by means of logistic regression with a 95% confidence interval. Variables with a significance level of p<0.20 in the bivariate analysis were incorporated into the multivariable regression model. Variables attaining a p-value less than or equal to 5% were considered statistically significant.

### Ethical issues

The project was approved by the Research Ethics Committee of the Santa Casa de Misericórdia de São Paulo (CAAE 05585518.7.0000.5479; opinion n°: 3.126.815 – 30/01/2019), as well as by other participating institutions. All participants signed an Informed Consent Form to participate in the study.

## RESULTS

A total of 1,317 transgender women and *travestis* were interviewed in the study. Of the total TGW interviewed, 698 (53%) suffered sexual violence during their lives, and, of 186, the violence occurred during their first sexual intercourse. Concerning the total sample (Table 2), the majority of TGW were aged between 18 and 29 years old (46.5%), were of black or brown race/color (70.8%), had eight to 11 years of study (54.2 %), and had an income of up to one BMW (49.5%). At the time of the interview, 41.4% of those interviewed declared that they were sex workers, and 73.4% had had commercial sex in their lives. In the 12 months prior to the study, 55.2% of respondents used some illicit drug.

**Table 2:** Characteristics of the sexual violence suffered by transgender women and *travestis* in Brazil, stratified by age.

Variable	Total (n=698)	18–29 years (n=320)	30–39 years (n=219)	=40 (n=152)	p-value
Frequency					
One time	232 (35,6)	125 (40,7)	67 (32,2)	40 (29,4)	0,033
More than one	419 (64,4)	182 (59,3)	141 (67,8)	96 (70,6)	
Perpetrator					
Unknown	235 (36,4)	120 (39,6)	66 (32,1)	49 (35,8)	0,252
Daily life	214 (33,1)	88 (29,0)	79 (38,3)	47 (34,3)	
Intimate	197 (30,5)	95 (31,4)	61 (29,6)	41 (29,9)	
Sought any health service					
Yes	47 (6,8)	28 (8,7)	15 (6,8)	4 (2,6)	0,048
No	648 (93,2)	294 (91,3)	205 (93,2)	149 (97,4)	
Reported it					
Yes	42 (6,0)	21 (6,5)	14 (6,4)	7 (4,6)	0,687
No	653 (94,0)	301 (93,5)	206 (93,6)	146 (95,4)	
Sought support from family					
Yes	94 (13,5)	50 (15,5)	28 (12,7)	16 (10,5)	0,293
No	601 (86,5)	272 (84,5)	192 (87,3)	137 (89,5)	
Sought any type of help					
Yes	146 (21,0)	77 (23,9)	45 (20,5)	24 (15,7)	0,117
No	549 (79,0)	245 (76,1)	175 (79,5)	129 (84,3)	

Note: Differences in numbers are due to missing values.

There were statistically significant differences between the groups of TGW who suffered or did not suffer sexual violence with the following variables: education (p=0.025), place of residence (p=0.030), use of illicit drugs (p<0.001) and who declared a state of health (p=0.011) and emotional health (p<0.001) as regular, bad or very bad. TGW who were sex workers at the time of the interview (p=0.001) or had already exchanged sex for money during their lives (p<0.001) showed a higher percentage of sexual violence.

Of the total number of TGW who experienced sexual violence, 64.4% reported that the violence was not a single episode, occurring on more than one occasion (Table 1). In the case of the most recent sexual violence, the most frequent perpetrators were unknown (36.4%). Regarding post-violence coping strategies, the majority of TGW did not seek a health service (93.2%), did not report it to police (93.9%), and did not seek support from family or friends (86.5%). TGW aged 40 or over had a higher percentage of violence perpetrated more than once when compared to younger ones (70.6 and 59.3%, respectively; p-value=0.033). The search for a health service after sexual violence was more prevalent among young TGW when compared to older TGW (8.8 and 2.6%, respectively; p-value=0.048).

In the univariate analysis (Table 3), variables such as having an income below the minimum wage, lacking stable housing, engaging in sex work or exchanging sex for money at any point in one’s life, using illegal drugs, self-assessing health status as regular/bad/very bad, perceiving emotional health negatively, and experiencing difficulties accessing health services were all correlated with a higher prevalence of sexual violence.

**Table 3:** Bivariate and multivariate logistic regression examining correlates of sexual violence suffered by transgender women and *travestis* women in Brazil.

Variable	PR (95%CI)	p-value	aPR (95%CI)	p-value
Age group				
18 to 29	1	0,379		
30 to 39	1.11 (0.86–1.44)			
=40	0.90 (0.68–1.19)			
Race/skin color				
White	1	0,792		
Non-white	1.10 (0.81–1.33)			
Education (years)				
Less than 12	0.75 (0.57–0.98)	0,038	0.59 (0.43–0.83)	0.002
=12	1		1	
Income (BMW)*				
=1	1.28 (1.02–1.60)	0,037	1.27 (0.98–1.64)	0.067
>1	1		1	
Living conditions				
Having a stable place to live	1	0,031	1	0.046
Not having a place to live	1.59 (1.05–2.42)		1.69 (1.01–2.84)	
Commercial sex (life)				
Yes	2.10 (1.64–2.69)	<0,001	2.04 (1.46–2.85)	<0,001
No	1		1	
Sex worker (current)				
Yes	1.62 (1.29–2.02)	<0,001	1.08 (0.80–1.46)	0,597
No	1		1	
Illegal drugs (12 months)				
Yes	1.50 (1.20–1.86)	<0,001	1.24 (0.96–1.60)	0,107
No	1		1	
Challenges to access health services (12 months)				
No	1	<0,001	1	<0,001
Yes	2.99 (1.99–4.47)		2.78 (1.74–4.43)	
Self-declared health status				
Good/very good	1	0,015	1	0,533
Regular/bad/very bad	1.35 (1.06–1.71)		0.91 (0.68–1.21)	
Self-declared emotional health				
Good/very good	1	<0,001	1	<0,001
Regular/bad/very bad	1.77 (1.41–2.21)		1.67 (1.28–2.19)	

Note: PR: prevalence ratio; aPR: adjusted prevalence ratio; CI: confidence intervals; BMW: Brazilian minimum wage.

* Brazilian minimum wage at the time (R$ 1,045.00, approximately 217.15 dollars).

In the adjusted analysis, the following factors remained associated with a higher prevalence of sexual violence: lacking stable housing (adjusted prevalence ratio — aPR=1.69, 95% confidence interval — 95%CI 1.01–2.84); exchanging sex for money during their lifetime (aPR=2.04, 95%CI 1.46–2.85); encountering difficulties in accessing health services (aPR=2.78, 95%CI 1.74–4.43); and rating their emotional health as regular, bad, or very bad (aPR=1.67, 95%CI 1.28–2.19). Interestingly, education exhibited unexpected behavior in both analyses, where lower education emerged as a protective factor against sexual violence (aPR=0.59, 95%CI 0.43–0.83).

## DISCUSSION

Violence is a serious public health issue that disproportionately affects transgender women globally. Our data indicate that transgender women and *travestis* experience a high prevalence of sexual violence (53%) throughout their lives, often beginning with their first sexual encounter and often occurring on more than one occasion. This prevalence contrasts sharply with the average prevalence of sexual violence among cisgender women in Brazil, which stands at 26.4%^22^. Additionally, our findings reveal that sexual violence among TGW is associated with factors such as inadequate housing, engaging in transactional sex at some point in life, difficulties accessing healthcare services, and poorer emotional well-being.

The characteristics of the most recent incident of sexual violence highlight that TGW often fall victim to violence perpetrated by multiple assailants. Notably, close to a third of the aggressors were individuals close to the victim, such as family members, friends, or partners. Intimate partner violence is prevalent in the transgender population, placing them at a higher risk compared to cisgender individuals^23^. In a relationship, the pre-existing trust can be manipulated to perpetuate abuse, resulting in a cycle of violence that is difficult to interrupt. Studies have indicated that intimate partner sexual violence is associated with higher levels of anxiety, depression, and abusive use of alcohol and illicit substances in transgender women^24,25^. Although we did not specifically assess intimate partner violence, in our study emotional health was also associated with sexual violence.

The elevated prevalence of violence during the first sexual encounter suggests that the perpetrators are individuals in whom the victim places trust. This form of violence may stem from the stigma that TGW individuals endure from the onset of their gender transition, as documented in other studies on the subject^26,27^. Additionally, the limited number of TGW seeking support from friends or family in coping with such violent situations underscores the lack of a robust support network.

The limited number of women who sought institutional help may indicate a lack of trust in the reliability of public institutions during this critical period. Research has consistently shown that TGW face discrimination and mistreatment by health professionals^28,29^. For instance, findings from the US Transgender Survey (USTS) indicate that 23% of respondents refrained from seeking medical attention when needed due to the fear of mistreatment based on their transgender identity^3^. Among those who did consult a healthcare professional in the past year, 33% reported encountering negative experiences related to their transgender status, including verbal harassment, refusal of treatment, or the necessity to educate healthcare providers about transgender issues to receive appropriate care^3^. Similar challenges are observed in police stations. Numerous studies illustrate that victims of sexual violence face disbelief and mistreatment within the justice system, starting from their initial interaction with law enforcement, where they undergo degrading police interrogations and uncomfortable genital examinations. This mistreatment persists through the resolution of the case before a judge, who may, unfortunately, publicize the identity of the victim or assign blame^7,11,12^. Recognizing that one of the primary hurdles in addressing sexual violence lies in comprehending the full scope of the problem, our data underscore the necessity for institutional efforts to promote fundamental health and citizenship rights for this vulnerable population.

Our findings indicate a heightened occurrence of sexual violence among TGW who are homeless or reside in shelters. The stigma and discrimination experienced by transgender people from a young age frequently compel them to leave their homes, intensifying their housing insecurity. When devoid of stable housing and lacking a support network, engaging in commercial sex is often perceived as an alternative means of income for survival^28^. Consistent with existing literature, our data underscore that TGW involved in the practice of commercial sex are even more susceptible to sexual and physical assaults^29-31^.

Our study yielded unexpected results, notably that low education emerged as a protective factor against sexual violence. This outcome may be linked to the nuanced perception of sexual violence among TGW. Low education levels can be indicative of greater vulnerability within this group, exposing them to various forms of violence, challenges in securing employment, and housing instability^32^. Consequently, sexual violence may become normalized and not readily identified as such, given its integration into the routine of prostitution or engaging in sex for some form of benefit.

The profile of the TGW studied highlights a context of elevated social vulnerability^17^. These individuals exhibit low levels of education and income, and engagement in prostitution is prevalent, accompanied by frequent drug use. In such a scenario, sexual violence may be exacerbated, leading to heightened physical and psychological consequences for the victims^32,33^.

The present study is subject to several limitations that warrant consideration. The cross-sectional design methodology precludes the establishment of a causal relationship between sexual violence and its consequences for victims, as well as the determination of a temporal relationship between the outcome and the variables under scrutiny. Additionally, the occurrence of potential biases stemming from self-reporting may influence the prevalence of the outcome. Moreover, given the utilization of the RDS method, we cannot assert that the sample is representative of the broader population of TGW in Brazil. Although the study outcome focuses on lifetime experiences of violence, it is important to note that the study was conducted during the early stages of the COVID-19 pandemic. This timing may have exacerbated prevalence estimates of violence, as studies indicate a higher frequency of domestic violence during this period^34,35^. Additionally, pandemic-related restrictions may have limited access to institutional support services for this population^35^.

The evidence presented in the study underscores the pressing need to address sexual violence against transgender women and *travestis*. The complexity of these experiences calls for a multifaceted response. Effectively preventing such violence relies on the development of public policies that fundamentally address transphobia, particularly at the institutional level. It is imperative to engage managers and institutions in education, health, and safety to implement gender-sensitive and culturally competent support systems catering to the specific needs of this population. Furthermore, the substantial gap in quantitative research and the absence of robust data emphasize the necessity for sustained investment in this area.
