# Supplementary material for: Lifetime sexual violence among transgender women and travestis (TGW) in Brazil: Prevalence and associated factors
Source: Rev Bras Epidemiol. 2024 Aug 19;27(Suppl 1):e240013.supl.1. doi: 10.1590/1980-549720240013.supl.1 (PMC11338532; doi:10.1590/1980-549720240013.supl.1)
Supplement: Translation

# Translation: Violência sexual durante a vida em mulheres trans e travestis (MTT) no Brasil: Prevalência e fatores associados

**DOI:** 10.1590/1980-549720240013.supl.1.2

**Authors:** Bruna Hentges, Rafael Steffens Martins, Jonatan da Rosa Pereira da Silva, Dariana Pimentel Gomes Hübner, Andréa Fachel Leal, Luciana Barcellos Teixeira, Daniela Riva Knauth, Maria Amélia de Sousa Mascena Veras

**Affiliations:** I Universidade Federal do Rio Grande do Sul, Programa de Pós-Graduação em Epidemiologia – Porto Alegre (RS), Brasil.; II Universidade Federal do Rio Grande do Sul, Departamento de Sociologia – Porto Alegre (RS), Brasil.; III Universidade Federal do Rio Grande do Sul, Departamento de Saúde Pública – Porto Alegre (RS), Brasil.; IV Santa Casa de São Paulo, Faculdade de Ciências Médicas – São Paulo (SP), Brasil.

**Keywords:** Mulher transgênero, Violência sexual, Vulnerabilidade social

## Abstract

**Objetivo:**

Descrever a prevalência, características e fatores associados à violência sexual em mulheres trans e travestis (MTT) no Brasil.

**Métodos:**

Estudo transversal conduzido em cinco cidades brasileiras (Campo Grande, Manaus, Porto Alegre, Salvador e São Paulo) entre 2019 e 2021. As participantes foram recrutadas usando a técnica *respondent-driven sampling* (RDS). O desfecho é a experiência de violência sexual ao longo da vida. As ações tomadas pelas vítimas e como elas lidaram com a experiência foram avaliadas. Análise de regressão logística foi empregada para examinar as associações entre fatores sociodemográficos e comportamentais (como raça, renda, trabalho sexual e acesso aos serviços de saúde) e o desfecho.

**Resultados:**

Um total de 1.317 MTT foram entrevistadas. Entre elas, 53% (n=698) relataram violência sexual. Para 64,4% (n=419) destas, a violência sexual ocorreu em mais de uma ocasião. A maioria das MTT não procurou serviços de saúde (93,2%, n=648), não denunciou (93,9%, n=653) nem buscou apoio de familiares ou amigos (86,5%, n=601). A maior prevalência de violência sexual foi associada à falta de moradia (razão de prevalência ajustada — RPa=1,69, IC 95% 1,01-2,84), histórico de envolvimento em trabalho sexual (RPa=2,04, IC 95% 1,46-2,85), relato de saúde emocional regular, ruim ou muito ruim (RPa=1,67, IC 95% 1,28-2,19) e experiência de dificuldades de acesso aos serviços de saúde (RPa=2,78, IC 95% 1,74-4,43).

**Conclusão:**

A alta prevalência de violência sexual, analisada em conjunto com as ações das vítimas, indica um contexto de alta vulnerabilidade e baixo suporte institucional. Nesse cenário, a violência pode ser exacerbada, resultando em graves consequências para a saúde.

## INTRODUÇÃO

A violência sexual é uma violação dos direitos humanos, com profundas consequências físicas, psicológicas e sociais para as suas vítimas. A violência sexual é um termo abrangente que inclui diferentes tipos de coerção, desde pressão social e intimidação até força física e penetração forçada^1^. Entre pessoas cisgênero (indivíduos cujo gênero está alinhado com o sexo atribuído ao nascimento), a violência sexual afeta de forma desproporcional as mulheres, com uma prevalência estimada ao longo da vida de 35,6%^1^.

Para indivíduos trans, aqueles cujo gênero difere daquele atribuído ao nascimento^2^, os estudos existentes indicam consistentemente uma maior prevalência de violência sexual, juntamente com outras formas de violência. No *US Transgender Survey* (USTS) de 2015, o estudo mais abrangente realizado com indivíduos trans até o momento (n=27.715), 47% de todos os entrevistados (homens e mulheres trans) relataram ter sofrido violência sexual durante a vida^3^. Especificamente para as mulheres trans, a prevalência da violência sexual ao longo da vida varia entre diferentes regiões do mundo: no USTS, a prevalência para esse grupo foi de 37%^3^; em um estudo abrangendo cinco países africanos, atingiu 54,4%^4^; e, em um estudo realizado no Haiti, a prevalência relatada foi de 75,5%^5^.

O número de estudos que investigam esse fenômeno aumentou substancialmente nos últimos anos. No entanto, uma proporção significativa deles examina pessoas trans como um grupo único, sem distinguir entre homens e mulheres trans, ou até mesmo apenas incorporando-os sob o guarda-chuva da identidade “LGBT”. Essa abordagem tende a homogeneizar as experiências de homens e mulheres trans, obscurecendo os vários estressores psicossociais únicos de cada identidade de gênero^6^. Nesse sentido, o presente estudo teve como objetivo descrever a prevalência, as características e os fatores associados à violência sexual em uma amostra de mulheres trans e travestis.

As mulheres trans são frequentemente vítimas de violência sexual devido a sua identidade e expressão de gênero (as maneiras como uma pessoa comunica seu gênero), que são percebidas como desviantes das normas e expectativas sociais^7,8^, e desafiam as dinâmicas de poder estabelecidas em uma sociedade heteronormativa^7,10^ e das autoridades policiais^11,12^, amplificando os desafios enfrentados pelas mulheres trans.

A violência, em suas diferentes formas, exerce uma influência adversa na saúde e bem-estar das mulheres trans, não apenas por meio de danos físicos imediatos, mas também pela intensificação do sofrimento psicológico^6^. Estudos têm demonstrado que a violência perpetrada contra mulheres trans está associada a diferentes resultados adversos para a saúde, como transtornos depressivos^13^, ansiedade^14^, ideação suicida^15^ e uso de substâncias ilícitas^16^. A violência sexual acrescenta ainda mais vulnerabilidade^17^ à vida das mulheres trans, que já é permeada por uma complexa cadeia de iniquidades sociais e de saúde.

A violência em sua manifestação mais grave, o homicídio, apresenta uma prevalência preocupante no contexto da América Latina. Organizações não governamentais dentro da comunidade LGBTQIA+, dedicadas a monitorar estatísticas globais sobre violência perpetrada contra pessoas trans, relatam que 73% desses homicídios ocorrem em territórios latino-americanos^18^. O Brasil, especificamente, surge como o principal *locus* de incidentes de homicídios de pessoas trans, sendo responsável por 31% de todas essas ocorrências em todo o mundo durante o período de 2022 a 2023^18^. Nesse contexto, é necessário compreender a extensão do problema da violência perpetrada contra mulheres trans e travestis no país.

Na região latino-americana, o termo “travesti” é frequentemente adotado por mulheres trans cujas trajetórias são caracterizadas por extrema vulnerabilidade, sendo a prostituição a principal forma de subsistência^19^. Com o objetivo de preservar a contextualização política e não negligenciar o histórico de marginalização social e econômica enfrentada pelas travestis, o presente estudo optou por utilizar o termo “mulheres trans e travestis” (MTT) para se referir ao grupo em análise.

## MÉTODOS

Os dados analisados neste artigo foram obtidos a partir da pesquisa transversal intitulada “Estudo sobre Prevalência de Sífilis e outras Infecções Sexualmente Transmissíveis entre Travestis e Mulheres Transexuais no Brasil: Cuidado e Prevenção” (TRANS Odara), realizada em cinco capitais brasileiras: Porto Alegre, São Paulo, Campo Grande, Salvador e Manaus. Como se trata de um grupo de difícil acesso por meio de técnicas tradicionais de amostragem, foi utilizada a técnica de amostragem por indicação de respondentes (*respondent-driven sample* – RDS), segundo a qual os participantes convidavam pessoas de sua rede de relacionamentos para participar do estudo^20^.

Os critérios de elegibilidade estabelecidos para participação no estudo foram: ter idade igual ou superior a 18 anos; ter sexo masculino designado ao nascer; identificar-se como travesti, mulher transexual ou qualquer outra autodesignação que implicasse uma identidade de gênero transfeminina; residir, trabalhar ou estudar nas cidades incluídas no escopo do estudo; e apresentar cupom de indicação fornecido por recrutador conhecido, seguindo a metodologia de amostragem RDS. Os critérios de exclusão foram estar sob efeito de álcool ou substâncias psicoativas de forma que impossibilitasse a entrevista.

A coleta de dados ocorreu entre dezembro de 2019 e julho de 2021. O estudo começou com uma fase formativa, utilizando diversos métodos de pesquisa, como o mapeamento de espaços sociais. As sementes do estudo foram identificadas através de entrevistas em profundidade e grupos focais dentro da comunidade de mulheres trans e travestis. Um tamanho de amostra de 1.280 mulheres trans e travestis foi determinado para estimar a prevalência da sífilis em cada local. Apesar dos desafios relacionados à pandemia que dificultaram o treinamento presencial, os investigadores com experiência anterior em estudos RDS facilitaram o treinamento e supervisão *online* para todas as atividades de pesquisa. Os participantes do estudo preencheram um questionário abrangendo informações sociodemográficas, comportamento sexual, abuso de substâncias e experiências de violência. Além disso, os participantes foram submetidos a testes rápidos para HIV, sífilis, hepatite B e hepatite C, e forneceram amostras de sangue, urina e secreções para exames laboratoriais posteriores. Maiores detalhes sobre o desenho da pesquisa podem ser encontrados em outro artigo publicado anteriormente^21^.

### Variáveis

O desfecho de interesse do estudo é a experiência de violência sexual ao longo da vida. A variável de desfecho foi criada a partir de duas questões distintas: se, em algum momento da vida, a entrevistada foi forçada a ter relações sexuais (sim/não) e se a primeira relação sexual foi forçada.

Variáveis sociodemográficas e comportamentais foram usadas para descrever a amostra e comparar grupos. A idade foi categorizada em grupos (18 a 29, 30 a 39 e 40 anos ou mais). A raça/cor foi autodeclarada, e categorizada em: branca, preta (incluindo pretas e pardas) e amarela/indígena. A escolaridade foi categorizada com base nos anos de estudo: menos de oito (correspondente ao ensino fundamental incompleto), oito a 11 (correspondente ao ensino fundamental completo/ensino médio incompleto) e 12 anos ou mais (ensino médio completo ou superior). A variável renda foi categorizada com base no salário mínimo brasileiro (SM) em vigor na época do estudo (R$ 1.045,00, aproximadamente 217,15 dólares). Para análise de regressão logística, as seguintes variáveis foram dicotomizadas: raça/cor (branca e não branca), escolaridade (menos de 12 anos de estudo e 12 anos ou mais), renda (até um SM ou mais de um), e problemas no acesso aos serviços de saúde nos últimos 12 meses (sim e não).

As entrevistadas que declararam viver em casa própria, alugada ou temporariamente com amigos/familiares foram categorizados como “tendo um lugar estável para morar”. Aquelas que declararam viver nas ruas, em uma pensão ou onde trabalhavam foram consideradas “não tendo um lugar para morar”. O sexo comercial foi avaliado com base na pergunta: “Você já fez sexo em troca de dinheiro, bens, drogas ou um lugar para morar?”. A variável “profissional do sexo” foi construída a partir de duas questões: ocupação principal no momento do estudo e declaração de que a principal fonte de renda no último mês provinha dessa ocupação. O uso de drogas ilícitas foi avaliado nos últimos doze meses e incluiu maconha (droga considerada ilegal no Brasil). As respostas sobre o estado de saúde no momento do estudo e a saúde emocional das participantes foram dicotomizadas. A avaliação das dificuldades de acesso aos serviços de saúde foi enquadrada pela seguinte pergunta: “No último ano, você encontrou algum desafio para acessar serviços de saúde relacionados à sua identidade de gênero?”.

Para as entrevistadas vítimas de violência sexual, avaliou-se a frequência da violência sexual (uma vez ou mais de uma vez) e o autor da última violência sofrida. O agressor foi categorizado como pessoa desconhecida, alguém do cotidiano da vítima (como um vizinho, atendente, cliente ou chefe) e alguém íntimo da vítima (amigo, membro da família ou companheiro). Foram verificados os recursos utilizados pela vítima após a última violência para lidar com o ocorrido: se procurou algum serviço de saúde, registrou queixa em uma instituição formal (como a polícia) e buscou apoio de familiares ou amigos. Os três recursos foram considerados para a variável “buscou qualquer tipo de ajuda”.

Considerando que o desfecho do estudo é a experiência de violência sexual ao longo da vida, levantou-se a hipótese de que mulheres trans e travestis mais velhas possam ter uma maior prevalência de tais experiências devido à sua maior exposição ao longo do tempo. Portanto, decidiu-se investigar essa relação, bem como se a idade se correlaciona com padrões variados de busca de assistência entre mulheres trans e travestis. Consequentemente, estratificou-se a Tabela 1 por faixa etária para explorar mais a fundo essas associações.

**Tabela 1:** Características sociodemográficas e comportamentais de mulheres trans e travestis no Brasil, estratificadas por violência sexual.

Variável	Violência sexual			
	Amostra total (n=1.317)	Sim (n=698, 53%)	Não (n=619, 47%)	Valor de p
Idade				
18 a 29	603 (46,5)	322 (53,4)	281 (46,6)	0,379
30 a 39	392 (30,2)	220 (56,1)	172 (43,9)	
=40	301 (23,2)	153 (50,8)	148 (49,2)	
Raça/cor da pele				
Branca	336 (25,7)	176 (52,4)	160 (47,6)	0,943
Negra	924 (70,8)	494 (53,5)	430 (46,5)	
Amarela/indígena	45 (3,5)	24 (53,3)	21 (46,7)	
Escolaridade (anos)				
<8	328 (25,0)	182 (55,5)	146 (44,5)	0,025
8 a 11	712 (54,2)	354 (49,7)	358 (50,3)	
=12	273 (20,8)	160 (58,6)	113 (41,4)	
Renda (SM)*				
<1	593 (49,5)	341 (57,5)	252 (42,5)	0,084
1 a 2	424 (35,4)	214 (50,5)	210 (49,5)	
>2	180 (15,1)	97 (53,9)	83 (46,1)	
Condições de moradia				
Tem moradia estável	1.166 (92,0)	608 (52,1)	558 (47,9)	0,030
Não tem moradia estável	101 (8,0)	64 (63,4)	37 (36,6)	
Sexo transacional (na vida)				
Sim	947 (73,4)	557 (58,0)	404 (42,0)	<0,001
Não	344 (26,6)	141 (39,6)	215 (60,4)	
Sexo transacional (atual)				
Sim	535 (41,4)	324 (60,0)	216 (40,0)	0,001
Não	756 (58,6)	374 (48,1)	403 (51,9)	
Drogas ilícitas (12 meses)				
Sim	727 (55,2)	418 (57,5)	309 (42,5)	<0,001
Não	590 (44,8)	280 (47,5)	310 (52,5)	
Dificuldade para acessar serviços de saúde (12 meses)				
Não, e procurou	758 (59,1)	392 (51,7)	366 (48,3)	<0,001
Não, mas não procurou	384 (30,0)	191 (49,7)	193 (50,3)	
Sim	140 (10,9)	106 (75,7)	34 (24,3)	
Condição de saúde autodeclarada				
Bom/muito bom	877 (68,6)	452 (51,5)	425 (48,5)	0,015
Regular/ruim/muito ruim	401 (31,4)	236 (58,9)	165 (41,1)	
Saúde emocional autodeclarada				
Bom/muito bom	650 (51,1)	304 (46,8)	346 (53,2)	<0,001
Regular/ruim/muito ruim	623 (48,9)	379 (60,8)	244 (39,2)	

As diferenças nos números são devidas a valores ausentes. SM: Salário mínimo brasileiro.

* Salário mínimo brasileiro na época (R$ 1.045,00, aproximadamente 217,15 dólares).

### Análise estatística

As análises foram realizadas utilizando o *software Statistical Package for Social Sciences* (SPSS, versão 22.0). As características da amostra foram descritas por N da categoria e percentual. As comparações entre grupos empregaram o teste de homogeneidade χ² de Pearson. Os fatores associados à violência sexual foram analisados por modelos bivariados e multivariados que estimaram as razões de prevalência por meio de regressão logística com intervalo de confiança de 95%. Variáveis com nível de significância p<0,20 na análise bivariada foram incorporadas ao modelo de regressão multivariada. As variáveis com valor de p menor ou igual a 5% foram consideradas estatisticamente significativas.

### Aspectos éticos

O projeto foi aprovado pelo Comitê de Ética em Pesquisa (CEP) da Santa Casa de Misericórdia de São Paulo (CAAE 05585518.7.0000.5479 - Nº parecer: 3.126.815 - 30/01/2019), assim como pelas demais instituições participantes. Todas as participantes assinaram o Termo de Consentimento Livre e Esclarecido para participar do estudo.

## RESULTADOS

Um total de 1.317 mulheres trans e travestis foram entrevistadas no estudo. Do total de MTT entrevistadas, 698 (53%) sofreram violência sexual durante a vida, sendo que para 186 destas, a violência ocorreu durante sua primeira relação sexual. Em relação à amostra total (Tabela 2), a maioria das MTT tinha entre 18 e 29 anos (46,5%), era da raça/cor preta ou parda (70,8%), tinha de oito a 11 anos de estudo (54,2%), e renda de até um SM (49,5%). No momento da entrevista, 41,4% das entrevistadas declararam ser profissionais do sexo e 73,4% já havia praticado sexo comercial na vida. Nos 12 meses anteriores ao estudo, 55,2% das entrevistadas usaram alguma droga ilícita.

**Tabela 2:** Características da violência sexual sofrida por mulheres trans e travestis no Brasil, estratificadas por idade.

Variável	Total (n=698)	18–29 anos (n=320)	30–39 anos (n=219)	=40 (n=152)	Valor de p
Frequência					
Uma vez	232 (35,6)	125 (40,7)	67 (32,2)	40 (29,4)	0,033
Mais de uma vez	419 (64,4)	182 (59,3)	141 (67,8)	96 (70,6)	
Agressor					
Desconhecido	235 (36,4)	120 (39,6)	66 (32,1)	49 (35,8)	0,252
Conhecido	214 (33,1)	88 (29,0)	79 (38,3)	47 (34,3)	
Íntimo	197 (30,5)	95 (31,4)	61 (29,6)	41 (29,9)	
Buscou serviços de saúde					
Sim	47 (6,8)	28 (8,7)	15 (6,8)	4 (2,6)	0,048
Não	648 (93,2)	294 (91,3)	205 (93,2)	149 (97,4)	
Reportou					
Sim	42 (6,0)	21 (6,5)	14 (6,4)	7 (4,6)	0,687
Não	653 (94,0)	301 (93,5)	206 (93,6)	146 (95,4)	
Buscou apoio da família					
Sim	94 (13,5)	50 (15,5)	28 (12,7)	16 (10,5)	0,293
Não	601 (86,5)	272 (84,5)	192 (87,3)	137 (89,5)	
Buscou algum tipo de ajuda					
Sim	146 (21,0)	77 (23,9)	45 (20,5)	24 (15,7)	0,117
Não	549 (79,0)	245 (76,1)	175 (79,5)	129 (84,3)	

Nota: As diferenças nos números são devidas a valores ausentes.

Houve diferenças estatisticamente significativas entre os grupos de MTT que sofreram ou não violência sexual em relação às seguintes variáveis: escolaridade (p=0,025), local de residência (p=0,030), uso de drogas ilícitas (p<0,001) e quem declarou estado de saúde (p=0,011) e saúde emocional (p<0,001) como regular, ruim ou muito ruim. As MTT que eram profissionais do sexo no momento da entrevista (p=0,001) ou que já haviam trocado sexo por dinheiro durante a vida (p<0,001) apresentaram maior percentual de violência sexual.

Do total de MTT que sofreram violência sexual, 64,4% relataram que a violência não foi um episódio único, ocorrendo em mais de uma ocasião (Tabela 1). No caso da violência sexual mais recente, os perpetradores mais frequentes eram desconhecidos (36,4%). Em relação às estratégias de enfrentamento pós-violência, a maioria das MTT não procurou um serviço de saúde (93,2%), não denunciou à polícia (93,9%) e não buscou apoio de familiares ou amigos (86,5%). MTTs com 40 anos ou mais apresentaram maior percentual de violência perpetrada mais de uma vez quando comparadas às mais jovens (70,6 e 59,3%, respectivamente; valor p=0,033). A procura por um serviço de saúde após violência sexual foi mais prevalente entre as MTT mais jovens quando comparadas às mais velhas (8,8 e 2,6%, respectivamente; valor de p=0,048).

Na análise univariada (Tabela 3), variáveis como ter renda inferior a um salário mínimo, não ter moradia estável, praticar trabalho sexual ou trocar sexo por dinheiro em algum momento da vida, usar drogas ilícitas, autoavaliar o estado de saúde como regular/ruim/muito ruim, perceber negativamente a saúde emocional e ter dificuldades para acessar serviços de saúde foram correlacionadas com uma maior prevalência de violência sexual.

**Tabela 3:** Regressão logística bivariada e multivariada examinando correlatos da violência sexual sofrida por mulheres trans e travestis no Brasil.

Variável	RP (IC 95%)	Valor de p	RPa (IC 95%)	Valor de p
Idade				
18 a 29	1	0,379		
30 a 39	1,11 (0,86–1,44)			
=40	0,90 (0,68–1,19)			
Raça/cor da pele				
Branca	1	0,792		
Não-branca	1,10 (0,81–1,33)			
Escolaridade (anos)				
Menos de 12	0,75 (0,57–0,98)	0,038	0,59 (0,43–0,83)	0.002
=12	1		1	
Renda (SM)*				
=1	1,28 (1,02–1,60)	0,037	1,27 (0,98–1,64)	0.067
>1	1		1	
Condições de moradia				
Tem moradia estável	1	0,031	1	0.046
Não tem moradia estável	1,59 (1,05–2,42)		1,69 (1,01–2,84)	
Sexo transacional (na vida)				
Sim	2,10 (1,64–2,69)	<0,001	2,04 (1,46–2,85)	<0,001
Não	1		1	
Sexo transacional (atual)				
Sim	1,62 (1,29–2,02)	<0,001	1,08 (0,80–1,46)	0,597
Não	1		1	
Drogas ilícitas (12 meses)				
Sim	1,50 (1,20–1,86)	<0,001	1,24 (0,96–1,60)	0,107
Não	1		1	
Dificuldade para acessar serviços de saúde (12 meses)				
Não	1	<0,001	1	<0,001
Sim	2,99 (1,99–4,47)		2,78 (1,74–4,43)	
Condição de saúde autodeclarada				
Bom/muito bom	1	0,015	1	0,533
Regular/ruim/muito ruim	1,35 (1,06–1,71)		0,91 (0,68–1,21)	
Saúde emocional autodeclarada				
Bom/muito bom	1	<0,001	1	<0,001
Regular/ruim/muito ruim	1,77 (1,41–2,21)		1,67 (1,28–2,19)	

Nota: RP: razão de prevalência; RPa: razão de prevalência ajustada; IC: intervalos de confiança; SM: Salário mínimo brasileiro.

* Salário mínimo brasileiro na época (R$ 1.045,00, aproximadamente 217,15 dólares).

Na análise ajustada, os seguintes fatores permaneceram associados a uma maior prevalência de violência sexual: falta de moradia estável (razão de prevalência ajustada — RPa=1,69, intervalo de confiança de 95% — IC95% 1,01–2,84); troca de sexo por dinheiro durante a vida (RPa=2,04; IC95% 1,46–2,85); encontrar dificuldades de acesso aos serviços de saúde (RPa=2,78; IC95% 1,74–4,43); e classificar a saúde emocional como regular, ruim ou muito ruim (RPa=1,67; IC95% 1,28–2,19). Curiosamente, a escolaridade apresentou um comportamento inesperado em ambas as análises, onde a menor escolaridade emergiu como um fator de proteção contra a violência sexual (RPa=0,59, IC95% 0,43–0,83).

## DISCUSSÃO

A violência é um grave problema de saúde pública que afeta desproporcionalmente as mulheres trans em todo o mundo. Nossos dados indicam que as mulheres trans e travestis sofrem uma elevada prevalência de violência sexual (53%) ao longo das suas vidas, que inicia na sua primeira relação sexual, e frequentemente ocorre em mais de uma ocasião. Essa prevalência contrasta fortemente com a prevalência média de violência sexual entre mulheres cisgênero no Brasil, que é de 26,4%^22^. Além disso, os resultados deste estudo revelam que a violência sexual entre as MTT está associada a fatores como moradia inadequada, envolvimento em sexo transacional em algum momento da vida, dificuldades de acesso aos serviços de saúde e pior bem-estar emocional.

As características da última violência sexual destacam que as MTT são muitas vezes vítimas de violência perpetrada por múltiplos agressores. Notavelmente, quase um terço dos agressores eram pessoas próximas da vítima, como familiares, amigos ou parceiros. A violência entre parceiros íntimos é prevalente na população trans, colocando-os em maior risco em comparação com indivíduos cisgênero^23^. Em um relacionamento, a confiança pré-existente pode ser manipulada para perpetuar o abuso, resultando em um ciclo de violência difícil de interromper. Estudos indicam que a violência sexual por parceiro íntimo está associada a níveis mais altos de ansiedade, depressão e uso abusivo de álcool e substâncias ilícitas em mulheres trans^24,25^. Embora não tenhamos avaliado especificamente a violência entre parceiros íntimos, no nosso estudo a saúde emocional também esteve associada à violência sexual.

A elevada prevalência de violência durante o primeiro encontro sexual sugere que os agressores são indivíduos em quem a vítima deposita confiança. Essa forma de violência pode decorrer do estigma que as MTT sofrem desde o início de sua transição de gênero, conforme documentado em outros estudos sobre o tema^26,27^. Além disso, o número limitado de MTT que procuram apoio de amigos ou familiares para lidar com situações violentas enfatiza a falta de uma rede de apoio robusta.

O número limitado de mulheres que buscaram ajuda institucional pode indicar uma falta de confiança nas instituições públicas durante este período crítico. Pesquisas têm consistentemente mostrado que as MTT enfrentam discriminação e maus-tratos por parte de profissionais de saúde^28,29^. Por exemplo, os resultados do USTS indicam que 23% dos entrevistados se abstiveram de procurar cuidados médicos quando necessário devido ao receio de maus-tratos com base na sua identidade trans^3^. Entre aqueles que consultaram um profissional de saúde no ano anterior, 33% relataram ter tido experiências negativas, incluindo assédio verbal, recusa de tratamento ou a necessidade de educar os profissionais de saúde sobre questões de gênero para receber cuidados adequados^3^. Desafios semelhantes são observados em delegacias de polícia. Numerosos estudos ilustram que as vítimas de violência sexual enfrentam descrença e maus-tratos no sistema judicial, desde a sua interação inicial com as autoridades policiais, onde são submetidas a interrogatórios policiais degradantes e a exames genitais desconfortáveis. Esses maus-tratos persistem através da resolução do caso perante um juiz, que pode, infelizmente, divulgar a identidade da vítima ou atribuir-lhe a culpa^7,11,12^. Reconhecendo que um dos principais obstáculos na abordagem da violência sexual reside na compreensão da dimensão total do problema, nossos dados destacam a necessidade de esforços institucionais para promover direitos fundamentais de saúde e cidadania para essa população vulnerável.

Nossos resultados indicam uma maior ocorrência de violência sexual entre MTT desabrigadas ou que residem em abrigos. O estigma e a discriminação vivenciados por pessoas trans desde jovens frequentemente as obrigam a deixar suas casas, intensificando sua insegurança habitacional. Quando desprovidas de moradia estável e sem uma rede de apoio, o envolvimento em sexo comercial é muitas vezes visto como um meio alternativo de renda para sobrevivência^29-31^.

Nosso estudo produziu resultados inesperados, como a baixa escolaridade, que emergiu como um fator de proteção contra a violência sexual. Esse resultado pode estar ligado à percepção diferenciada da violência sexual entre as MTT. Níveis baixos de escolaridade podem indicar uma maior vulnerabilidade deste grupo, expondo-as a diversas formas de violência, além de desafios na garantia de emprego e instabilidade habitacional^32^. Consequentemente, a violência sexual pode tornar-se normalizada e não ser facilmente identificada como tal, dada sua integração à rotina da prostituição ou da prática sexual em troca de algum tipo de benefício.

O perfil das MTT estudado evidencia um contexto de elevada vulnerabilidade social^17^. Essas mulheres apresentam baixos níveis de escolaridade e renda, e o envolvimento na prostituição é predominante, acompanhado do uso frequente de drogas. Em tal cenário, a violência sexual pode ser exacerbada, levando a maiores consequências físicas e psicológicas para as vítimas^32,33^.

O presente estudo está sujeito a várias limitações que merecem consideração. A metodologia de desenho transversal impede o estabelecimento de uma relação causal entre violência sexual e suas consequências para as vítimas, assim como a determinação de uma relação temporal entre o resultado e as variáveis em análise. Além disso, potenciais vieses decorrentes do autorrelato podem influenciar a prevalência do desfecho. Ainda, dada a utilização do método RDS, não podemos afirmar que a amostra seja representativa da população mais ampla de MTT no Brasil. Embora o desfecho do estudo se concentre em experiências de violência ao longo da vida, é importante observar que o estudo foi realizado durante as fases iniciais da pandemia de COVID-19. Esse momento pode ter exacerbado as estimativas de prevalência de violência, uma vez que estudos indicam maior frequência de violência doméstica durante esse período^34,35^. Por fim, as restrições relacionadas à pandemia podem ter limitado ainda mais o acesso aos serviços de apoio institucional para esta população^35^.

As evidências apresentadas no estudo destacam a necessidade urgente de abordar a violência sexual contra mulheres trans e travestis. A complexidade dessas experiências requer uma resposta multifacetada. Prevenir efetivamente tal violência depende do desenvolvimento de políticas públicas que abordem fundamentalmente a transfobia, especialmente no nível institucional. É imperativo envolver gestores e instituições em educação, saúde e segurança para implementar sistemas de apoio sensíveis ao gênero e culturalmente competentes, que atendam às necessidades específicas dessa população. Além disso, a lacuna substancial na pesquisa quantitativa e a ausência de dados robustos destacam a necessidade de investimento contínuo nesta área.
